# Determination of multiclass, semi-polar pesticide residues in fatty fish muscle tissue by gas and liquid chromatography mass spectrometry

**DOI:** 10.1016/j.mex.2019.04.014

**Published:** 2019-04-16

**Authors:** Marcos Colazzo, Beatriz Alonso, Federico Ernst, María Verónica Cesio, Andrés Perez-Parada, Horacio Heinzen, Lucía Pareja

**Affiliations:** a Departamento de Química del Litoral-DQL, CENUR Litoral Norte, Universidad de la República (UdelaR), Ruta 3 Km 363, 60000, Paysandú, Uruguay; b Grupo de Análisis de Compuestos Traza – GACT, Facultad de Química, Universidad de la República (UdelaR), General Flores 2124, 11800, Montevideo, Uruguay; c Departamento de Desarrollo Tecnológico – DDT, Centro Universitario Regional del Este (CURE), Universidad de la República (UdelaR), Ruta 9 y Ruta 15, 27000, Rocha, Uruguay

**Keywords:** QuEChERS (Quick, Easy, Cheap, Effective, Rugged and Safe), Fish tissue, Pesticide residues, QuEChERS, GC–MS, LC–MS/MS

## Abstract

With the aim of monitoring multiclass semi-polar pesticide residues in freshwater fatty fish, two QuEChERS approaches (so-called acetate buffered and unbuffered versions) were evaluated for the determination of 77 pesticide residues. Compounds were selected according to the dominant rainfed agriculture activities in South America. Unbuffered QuEChERS was finally chosen for validation purposes owing that it provided the best results in terms of recovery yields. Method performance was evaluated in two instrumental systems, liquid chromatography – tandem mass spectrometry (LC–MS/MS) in Scheduled MRM™ algorithm available on hybrid quadrupole – linear ion trap (QLIT) instrument, and gas chromatography – mass spectrometry (GC–MS) under selected ion monitoring (SIM) mode.

Spiking experiments were carried out to determine the trueness, precision, linearity, limit of quantification of the method as well as matrix effect.

The Unbuffered QuECHERS method described here:

• Was validated for the analysis of 67 pesticide residues in fish muscle tissue.
• Presented quantification limits in the range 1–15 μg kg^−1^ for the vast majority of the studied compounds.
• Enable environmental monitoring of pesticide residues in fish due to their low LOQs.

## Graphical abstract

**Figure:**
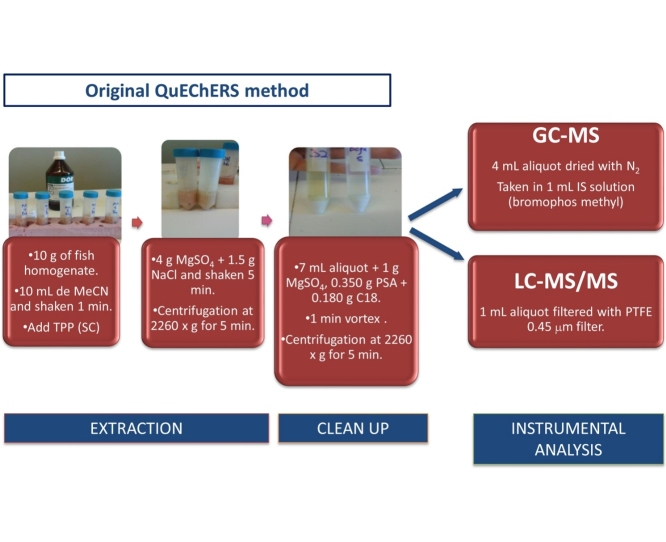

**Specifications Table**

**Table:** 

Subject area:	Environmental Sciences
More specific subject area:	Pesticide residues in biota
Method name:	QuEChERS (Quick, Easy, Cheap, Effective, Rugged and Safe)
Name and reference of original method:	Original (unbuffered) QuEChERS [1] Acetate buffered QuEChERS [2]
Resource availability:	Not applicable

## Method details

### Sample treatment

1) Frozen samples of Megaleporinus obtusidens fish muscle tissue (fillet) are chopped and homogenized with a stainless-steel kitchen cutter taking care of not unfreezing the sample. The fat content of the samples is 15 ± 2% (w/w) (n = 5) [3].
2) Homogenate is frozen again at −18 °C until analysis.

Unbuffered QuEChERS approach for the extraction of pesticide residues in fish [1,3,4]

1) Weight 10 g of frozen fish sample into a 50-mL centrifuge tube and add 10 mL of MeCN. Shake vigorously by hand during 1 min. Add 10 μL aliquot of 10 μg mL^−1^ of triphenyl phosphate (TPP) solution as surrogated compound (SC) and let stand 1 min.
2) Add 1.5 g of NaCl and 4 g of anhydrous MgSO_4_. Shake vigorously by hand during 4 min. Centrifuge at 2260 × *g* during 5 min.
3) Transfer 7 mL aliquot of the upper layer to a 15-mL tube containing 350 mg PSA, 180 mg of C-18 and 1 g of anhydrous MgSO_4_. Vortex the mix for 30 s and centrifuge it at 2260 × *g* for 5 min.
4) Filter 1 mL of supernatant through a 0.22 μm PTFE filter and collect into a 2-mL screw-cap vial for LC–MS/MS analysis.
5) Evaporate to dryness 4 mL of the cleaned-up solution under a gentle nitrogen stream. Re-dissolve in 1 mL of bromophos methyl (0.5 mg mL^−1^, internal standard (IS)) in EtOAc for GC–MS analysis. The equivalent tissue concentration per sample extract was 1 g mL^−1^ for LC–MS/MS and 4 g mL^−1^ for GC–MS.

Acetate buffered QuEChERS approach for the extraction of pesticide residues in fish [2,5]

1) Weight 10 g of frozen fish sample into a 50-mL centrifuge tube and add 10 mL of 1% Acetic acid in MeCN. Shake vigorously by hand during 1 min. Add 10 μL aliquot of 10 μg mL^−1^ of TPP (SC) solution and let stand 1 min.
2) Add 4 g of anhydrous MgSO_4_ and 1.5 g of NaAc·3 H_2_O. Shake vigorously by hand during 4 min. Centrifuge at 2260 × *g* during 5 min.
3) Transfer 7 mL aliquot of the upper layer to a 15-mL tube containing 350 mg PSA, 180 mg of C-18 and 1 g of anhydrous MgSO_4_. Vortex the mix for 30 s in and centrifuge it at 2260 × *g* for 5 min.
4) Filter 1 mL of supernatant through a 0.22 μm PTFE filter and collect into a 2-mL screw-cap vial for LC–MS/MS analysis.
5) Evaporate to dryness 4 mL of the cleaned-up solution under a gentle nitrogen stream. Re-dissolve in 1 mL of bromophos methyl (0.5 mg mL^−1^, IS) in EtOAc for GC–MS analysis. The equivalent tissue concentration per sample extract was 1 g mL^−1^ for LC–MS/MS and 4 g mL^−1^ for GC–MS.

### Gas chromatography–mass spectrometry (GC–MS)

A Shimadzu GC-QP2010 Ultra (Kyoto, Japan) equipped with Thermo Scientific (Waltham, MA, USA) TRACE™ TR-5MS (5% phenyl polysilphenylene-siloxane) bonded fused-silica capillary column (30 m × 0.25 mm i.d. × 0.25 mm film thickness). Electron impact ionization (EI) mass spectra was obtained at 70 eV and monitored from 50 to 550 *m*/*z* for full scan mode analysis. MS system was programmed in selected ion monitoring (SIM) mode. The working parameters were: injector temperature 280 °C;

interface temperature 280 °C; carrier gas He at 1 mL min^−1^. Oven conditions; from 120 °C initial (5 min hold), increased to 190 °C at a rate of 10 °C/min (1 min hold), then to 250 at 5 °C min (5 min hold), finally to 300 °C at 5 °C/min (5 min hold). Injection mode: splitless; injection volume: 1.0 μL. The identification of the compounds was confirmed by injection of solvent and matrix matched standards and comparison of their retention index and relevant MS ratios. GC–MS Solution version 4.11 SU2 with MS libraries was used for instrument control and data processing. Spectrometric conditions are detailed in Table S1.

### Liquid chromatography – tandem mass spectrometry (LC–MS/MS)

LC–MS/MS analysis were performed with an Agilent 1200 LC system (Agilent Technologies, Palo Alto, USA) coupled to a Sciex 4000QTRAP (Concord, Canada), quadrupole-linear ion trap, operated in triple quadrupole MS/MS mode. LC-Separation was performed on a ZORBAX Eclipse XDB-C18 (150 mm × 4.6 mm, 5 μm) column (Agilent Technologies, Santa Clara, CA, USA).

The operation of the LC gradient involved the following two eluent components: (A) water/formic acid 0.1% and (B) MeCN. It was run at 600 μL min^−1^ starting with 90% component A at injection time and stable for 3 min, gradually changing to 0% A (100% B) over 20 min and stable for 5 min, then to 90% A (10% B) over 3 min. This eluent composition was kept for 5 min, computing a total time of 33 min run. The injection volume was 5 μL. MS/MS detection was performed as previously reported [3]. Table S2 summarizes compound dependent parameters. Analyst software v 1.5.1 (SCIEX) was used for data acquisition and processing.

### Methods comparison

Firstly, both QuEChERS approaches (unbuffered and acetate buffered) were compared at 50 μg kg^−1^ level, in terms of % Rec and repeatability, for the seventy-seven compounds listed in Table S3.

Recovery results at 50 μg kg^−1^ showed that the performance of these methods was very similar.

**Fig. 1:**
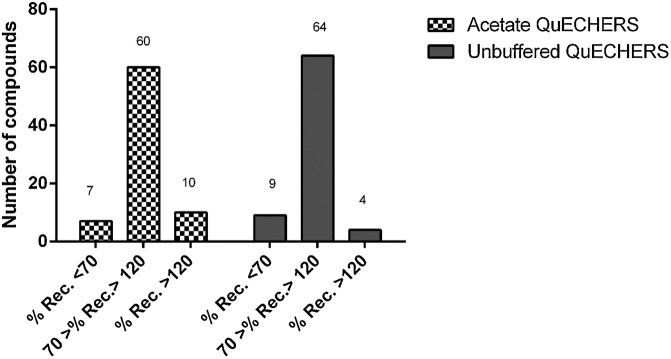
Comparison of the unbuffered and the buffered acetate QuEChERS.

Unbuffered QuEChERS method presented good recovery percentages for 64/77 pesticides whereas for the acetate buffered version, 60 compounds presented good performance. Therefore, the unbuffered QuEChERS was chosen for method validation (Fig. 1). In addition, as this fish matrix presents a relatively large amount of lipids (15 ± 2% (w/w)) C-18 was included for the d-SPE step.

Compounds in GC were quantified by IS method using bromophos methyl whereas external calibration was employed in the LC system. TPP was selected as a QA/QC strategy with the aim of generating control charts for the on-going validation process.

Three pesticides, iprodione, fenvalerate and tetradifon presented good results with the acetate version. However, they could not be recovered with the unbuffered approach and they were excluded from method validation. A possible explanation is that pH of the extract in the acetate buffered version improves their recovery, but in the unbuffered version, where the pH is around 8 the recovery of these compounds is affected. In the other way, azinphos methyl, malathion, methidathion and boscalid presented acceptable recoveries with the unbuffered approach while with the acetate version were not in the range 70–120%.

Some of the selected compounds, especially acidic herbicides (quinclorac, dicamba, bispyribac-sodium), some fungicides (chlorothalonil, folpet, spiroxamine) and the pyrethroid deltamethrin, presented recovery problems in both QuEChERS methods and they were finally excluded for subsequent experiments. A possible explanation for the low recoveries of the acidic compounds is the use of PSA. This adsorbent, a primary and secondary amine, can interact with acidic compounds yielding low recoveries after the clean-up step. Folpet is widely reported as a troublesome compound for GC–MS analysis because of its thermal degradation [6]. Moreover, it is reported that chlorothalonil presents recovery problems in some matrices when extracted with acetonitrile. Then, 67 pesticides that presented satisfactory preliminary results were included for method validation study.

### Method validation

The performance of the evaluated method was studied in terms of recoveries, limits of quantifications, precision, linearity and matrix effect according to SANTE guidelines [7].

The recoveries for the different assayed levels are represented in Table 1, Table 2 for GC–MS and LC–MS/MS, respectively.

**Table 1:** Recovery, repeatability, LOQ, ME and Ion ratios of the selected pesticides for GC–MS in SIM mode.

Target compound	15 μg kg^−1^		50 μg kg−^1^		100 μg kg^−1^		LOQ (μg kg^−1^)	ME (%)	Ion ratio (%)
	%Rec	%RSD	%Rec	%RSD	%Rec	%RSD			
*O*-phenylphenol	70	7	94	6	78	5	15	−25	77
Bromopropylate	65	>20	86	12	74	4	50	0.3	55
Buprofezin	66	7	86	5	79	5	50	−18	49
Chlorfenvinphos	76	18	90	5	88	5	15	−1.3	65
Chlorpyrifos	60	>20	75	4	76	7	50	−11	99
Chlorpyrifos methyl	58	5	88	4	75	3	50	−2.4	70
Coumaphos	59	35	121	7	72	10	50	108	73
Cyhalofop butyl	71	6	109	5	91	2	15	29	63
Cypermethrin	90	20	158	14	75	10	15	67	75
Diazinon	80	9	95	5	82	6	15	−19	65
Ethion	72	12	99	5	84	3	15	16	64
Fenhexamid	36	>20	123	17	73	6	50	140	53
Fenthion	50	18	96	5	68	16	50	5.4	34
Fipronil	92	5	112	5	94	4	15	0.2	68
Kresoxim methyl	86	7	101	3	87	4	15	−7.9	52
Parathion ethyl	105	>20	119	8	88	10	15	27	60
Parathion methyl	113	18	109	8	85	5	15	31	24
Pyriproxyfen	80	14	75	8	78	4	15	8.8	15
τ-fluvalinate	63	13	98	13	74	3	50	27	34
Trifluralin	70	3	85	6	78	4	15	−16	16
Vinclozolin	76	5	92	3	84	4	15	−9.5	90
β-cyfluthrin	45	>20	118	12	88	10	50	23	73
λ-cyhalothrin	76	11	98	9	84	4	15	10	62

**Table 2:** Recovery, repeatability, LOQ, ME and ion ratios of the selected pesticides for LC–MS/MS in MRM mode.

Pesticide	1.0 μg kg^−1^		10 μg kg^−1^		50 μg kg^−1^		LOQ (μg kg^−1^)	ME (%)	Ion Ratio (%)
	% Rec	% RSD	% Rec	% RSD	% Rec	% RSD			
Acetamiprid	106	2	93	1	108	5	1.0	−0.3	10
Ametryn	93	7	93	4	115	3	1.0	−0.1	18
Amitraz	68	3	67	2	78	3	1.0	−0.3	5
Atrazine	103	5	98	2	98	1	1.0	3.0	8
Azinphos methyl	83	17	88	17	110	15	1.0	24	32
Azoxystrobin	99	9	96	4	117	5	1.0	−16	8
Boscalid	--	--	96	4	105	5	10	−0.2	18
Carbaryl	87	7	99	4	96	5	1.0	−0.5	1
Carbendazim	93	9	91	1	141	2	1.0	−0.2	11
Carbofuran	89	2	93	19	117	8	1.0	−61	15
Clomazone	85	7	89	3	54	4	1.0	−12	47
Cyproconazole	90	5	100	3	100	1	1.0	−7.6	3
Difenoconazole	94	6	98	3	96	2	1.0	−1.7	18
Dimethoate	102	4	93	2	105	1	1.0	−31	46
Epoxiconazole	116	11	94	4	97	2	1.0	0.9	22
Flutriafol	94	10	96	3	102	2	1.0	−8.4	95
Flusilazole	103	7	99	6	104	4	1.0	−4.1	37
Hexythiazox	70	8	73	3	67	17	1.0	−64	42
Imazalil	--	--	85	20	87	5	10	−7.9	19
Malaoxon	108	7	93	4	105	3	1.0	−2.9	53
Malathion	82	7	99	2	100	2	1.0	−22	58
Metalaxyl	111	5	95	2	100	1	1.0	−13	48
Metamidophos	72	4	70	1	69	10	1.0	−44	50
Methidathion	88	8	108	7	99	1	1.0	−21	72
Methiocarb	133	14	96	10	81	1	10	−31	36
Metolachlor	94	5	92	1	126	4	1.0	−9.2	17
Metribuzin	122	11	94	3	120	5	1.0	−17	16
Metsulfuron methyl	92	8	74	9	106	7	1.0	−7.0	24
Pendimethalin	85	10	76	3	86	11	1.0	−40	7
Penoxulam	69	10	70	4	97	3	1.0	0.7	12
Pirimicarb	98	6	94	2	101	3	1.0	−14	76
Pirimiphos methyl	89	5	86	4	94	2	1.0	−4.4	66
Prochloraz	93	6	103	5	100	0	1.0	−2.6	9
Propanil	87	5	96	5	95	2	1.0	14	45
Propiconazole	100	6	90	2	98	3	1.0	5.8	53
Pyraclostrobin	90	5	95	2	98	7	1.0	−20	98
Pyrazosulfuron ethyl	75	8	82	4	89	7	1.0	4.4	10
Pyrimethanil	90	12	84	4	83	5	1.0	−17	89
Tebuconazole	98	6	97	2	93	2	1.0	1.1	2.0
Thiacloprid	99	5	97	5	103	17	1.0	−56	19
Thiabendazole	91	9	88	2	107	4	1.0	−28	47
Thiamethoxam	107	10	90	4	123	3	1.0	−71	31
Tricyclazole	88	5	95	3	120	3	1.0	−2.1	79
Trifloxystrobin	89	7	95	3	98	1	1.0	−32	41

As it is shown in Table 1, the GC-amenable compounds presented very good recoveries at 50 and 100 μg kg^−1^. However, at the lowest fortification level, out of the 26 pesticides, presented recovery problems, either below or above the range accepted by the DG SANTE [7].

For LC—MS/MS, thirty-seven compounds presented acceptable recoveries at all the spiked levels (%Rec. between 69 and 123) with repeatability, expressed as RSD%, in the range 2–20%. However, some compounds such as boscalid, carbendazim, clomazone, hexythiazox, imazalil, methiocarb, and metolachlor did not complied with the DG SANTE criteria in at least one of the evaluated levels (% Rec < 67 or % Rec > 123) (Table 2).

In this work only the parent compound amitraz was evaluated whereas its metabolites were not studied. This compound is very lipophilic (pKow 5.5), so it is potentially bioaccumulable. However, it is very unstable compound even during analysis [8]. As it is shown in Table 2, recoveries for amitraz were rounding the minimal acceptability criteria (70% for tested levels). Inclusion of amitraz metabolites should be studied in future studies. Fig. S1 shows the two transitions optimized for the analysis of this compound.

The LOQs along with recovery percentages are listed in Table 1, Table 2 for the selected compounds for both instrumental systems. LOQs were determined according SANTE criteria, as the lowest spiked level of the validation, meeting the method performance acceptability criteria. The LOQs obtained in the GC–MS analyses were 15 μg kg^−1^ for 16 compounds and 50 μg kg^−1^ for the rest of the pesticides (Table 1). For LC–MS/MS, 39 out of the 44 pesticides presented LOQs at 1 μg kg^−1^ while for boscalid, imazalil, methiocarb and penoxsulam at 10 μg kg^−1^ (see Table 2).

Linearity was evaluated in solvent and in matrix-matched calibration prepared respectively at five concentration levels. The calibration curves were constructed using least-squares regression from the injection of blank sample spiked with the standards. The fit of the linear calibration function in matrix and in solvent was inspected visually. Moreover, the residuals were calculated. The linearity along the studied range presented coefficients of determination higher than 0.99 for all target compounds.

Matrix effect, defined as signal suppression or enhancement, relative to analyte signal in solvent, is a major drawback for quantitative trace analysis by LC-ESI/MS and GC–MS systems. Matrix co-extractives can compromise the quantitative analysis of the compounds at trace levels, as well as it can greatly affect the method accuracy and reproducibility. Several proposals have been published to overcome this problem but the most common one is the use of matrix-matched calibration standards for the quantification of the target compounds [9,10].

**Fig. 2:**
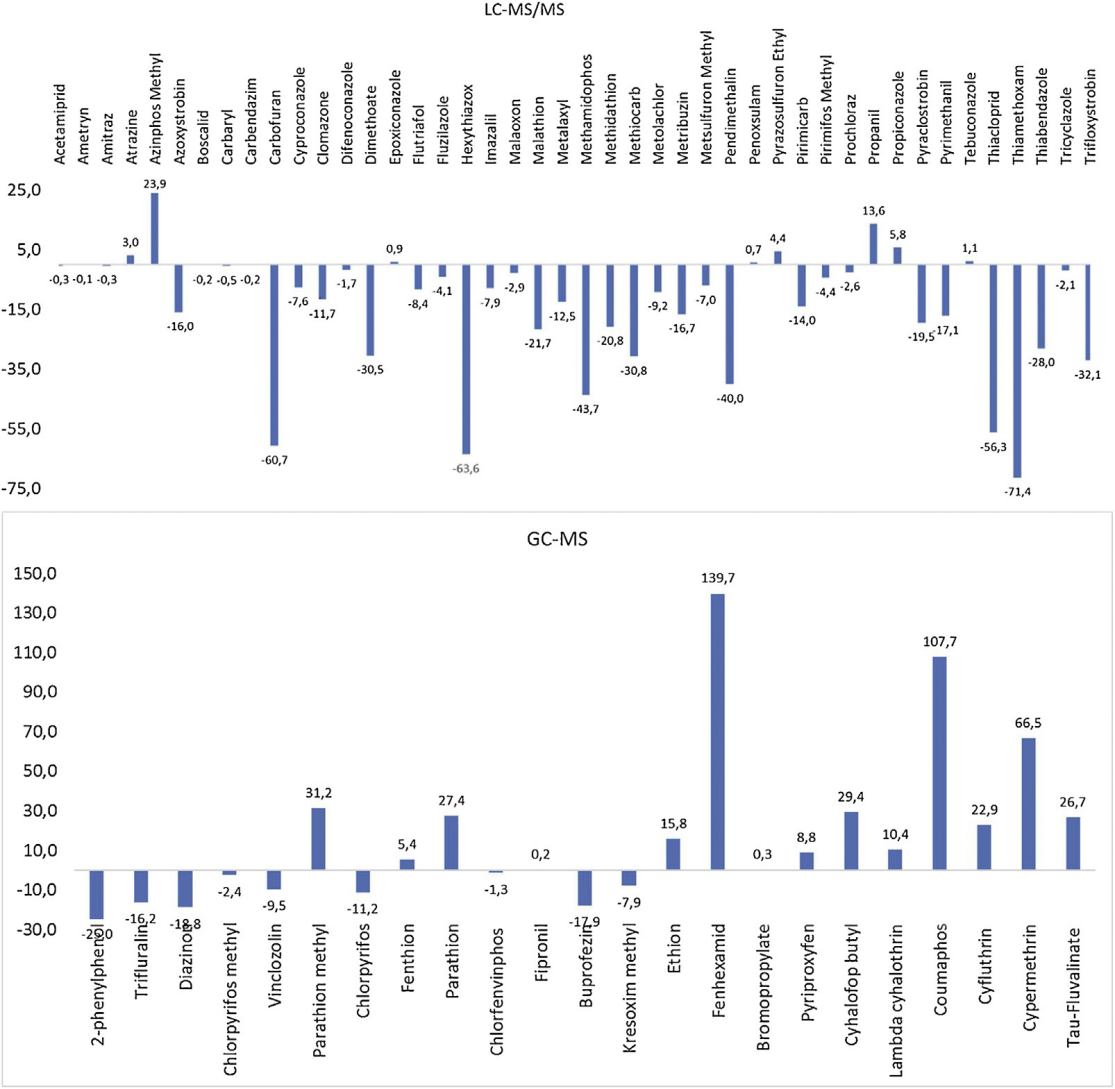
Matrix Effect for the evaluated compounds obtained by a. LC–MS/MS and b. GC–MS.

Depending on the value (in percentage), different matrix effects could be observed. Signal enhancement occurs if the percentage of the difference between the slopes is positive whereas a negative value it is indicative of signal suppression. A percentage between −20% and 20% was considered as no matrix effect. A medium matrix effect was observed when the values ranged between −50% and −20% or 20–50% and a strong ME would be below −50% or above 50% (Fig. 2) [10].

As shown in the Fig. 2a, for LC–MS/MS, practically all the compounds showed signal suppression. However, this effect was negligible except for four pesticides (carbofuran, hexythiazox, thiacloprid, thiamethoxam), that presented a strong effect, between 56 and 71% (Fig. 2a). Conversely, for GC–MS, only fenhexamid, cypermethrin and coumaphos presented a high signal enhancement (between 67 and 140%) (Fig. 2b).

On-going method verification trough within laboratory reproducibility (RSD_w_*_R_*) was evaluated for the TPP (SC). Average recovery was 100.6% with RSD_w_*_R_* of 16.5% (n = 149) [3].

### Identification criteria

The identification of the compounds was performed based on SANTE document [7] which establish retention time matching (±0.1 min), a minimum number of ions to be monitored (3 ions for SIM and 2 product ions for MS/MS based acquisitions), the analyte peaks in the extracted ion chromatograms must fully overlap and the ion ratio must be within ±30% (relative) of average of calibration standards from same sequence. Table 1, Table 2 show the different ion ratio obtained for all pesticides in both systems.

### Additional information

Fillet of fatty fish species showed few dispersion of fat content (˜15%) between different samples. In general, fish fillet is composed by ˜70% moisture and residual amount of proteins (approx. 15%) (unpublished data for Megaleporinus obtusidens). Other reports pointed out the importance of particular composition of matrices in method development [11].

Despite of the complexity of the sample, our results demonstrate that the methodology is suitable for analysis of trace concentrations of pesticide residues, enabling multi-class pesticide determination at low part per billion (ppb) levels. Alternative matrices aiming environmental monitoring other than those employed for human consumption are increasingly being used for pesticide testing. This methodology and levels assayed in recovery studies were intended for studies aiming dynamics of pesticides [12]. The method is particularly useful to ascertain the correspondence of pesticide findings fitting the technological package employed in South American rainfed agriculture.

Our results suggest the unbuffered QuEChERS as a suitable methodology for the determination of 67 different pesticide residues incorporating LC and GC amenable compounds. Selectivity and sensitivity was fit for the purpose. Clean-up step demonstrated suitability for routine application during more than 2 years without any additional system maintenance over other food matrices.

Recent methodologies looking for pesticide residues in fish muscle tissue have been reported aiming multi-class monitoring of pesticide residues. Particular advantages are demonstrated in high sample throughput and simplicity over multi-method approaches [13]. This study incorporates a list of GC amenable insecticides and fungicides with different phisicochemical properties (Table S3) [14]. On the other hand, citrate buffered QuEChERS with final PSA and C18 cleanup has been recently reported for the quantitative analysis of 44 pesticide residues in fish muscle by LC—MS/MS [15]. Additionally, that report used isotope dilution approach which is in practice limiting for most research laboratories in terms of analytical costs. In this work, we used only one IS for a group of 23 GC amenable compounds. Although linearity problems with bromophos methyl were not evidenced, some gap of improvement is desired for the use of a group of isotopically labeled internal standards covering different families of pesticides.
