# Supplementary material for: Determination of multiclass, semi-polar pesticide residues in fatty fish muscle tissue by gas and liquid chromatography mass spectrometry
Source: MethodsX. 2019 Apr 16;6:929–37. doi: 10.1016/j.mex.2019.04.014 (PMC6500907; doi:10.1016/j.mex.2019.04.014)
Supplement: Supplementary file 1 [file mmc1.doc]

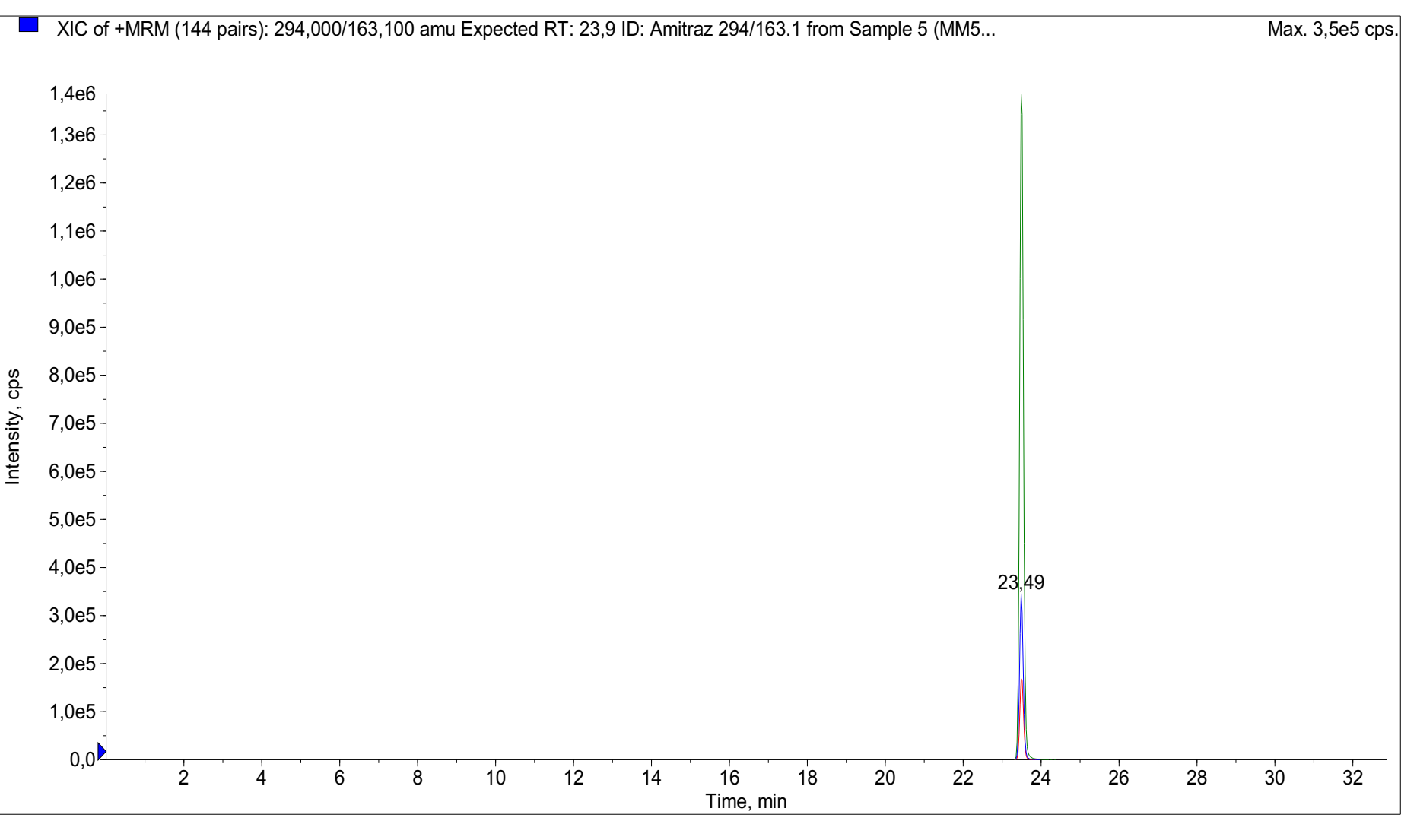


*Figure S1. Extracted ion chromatogram for amitraz in LC-MS/MS*

*Table S1: GC-MS analysis parameters of the target compounds.*

| ***Pesticide*** | ***t_R_ (min)*** | ***Quantifier***  ***m/z*** | ***Qualifiers***  ***m/z*** |
| --- | --- | --- | --- |
| *O-phenylphenol* | *11.56* | *170* | *169; 141* |
| *Bromophos methyl (IS)* | *19.63* | *333* | *331; 329* |
| *Bromopropylate* | *27.43* | *341* | *339; 183* |
| *Buprofezin* | *22.59* | *105* | *172; 175* |
| *Chlorfenvinphos* | *19.85* | *267* | *269; 323; 295* |
| *Chlorpyrifos* | *18.74* | *197* | *314; 258; 199* |
| *Chlorpyrifos methyl* | *17.24* | *286* | *288; 290* |
| *Coumaphos* | *33.34* | *226* | *362; 109; 364* |
| *Cyhalofop butyl* | *29.78* | *229* | *256; 357; 120* |
| *Cypermethrin* | *35.42; 35.50; 35.62; 35.75* | *163* | *181; 165* |
| *Diazinon* | *15.39* | *179* | *152; 304; 276* |
| *Ethion* | *23.79* | *231* | *203; 153; 384* |
| *Fenhexamid* | *23.79* | *97* | *177; 179* |
| *Fenthion* | *19.35* | *278* | *169, 125* |
| *Fipronil* | *19.98* | *367* | *369; 213* |
| *Kresoxim methyl* | *22.61* | *116* | *206; 131* |
| *Parathion ethyl* | *19.12* | *109* | *291; 139* |
| *Parathion methyl* | *17.56* | *109* | *263; 233* |
| *Pyriproxyfen* | *29.72* | *136* | *96; 78* |
| *τ-fluvalinate* | *37.63; 37.81* | *250* | *252; 181* |
| *Trifluralin* | *13.26* | *306* | *264; 290* |
| *Vinclozolin* | *17.33* | *212* | *285; 198* |
| *β-cyfluthrin* | *34.85; 35.19* | *163* | *226; 165; 206* |
| *λ-cyhalothrin* | *30.18* | *181* | *197; 208; 209* |

*Table S2. LC-MS/MS analysis parameters of the target compounds.*

| ***Pesticide*** | ***t_R_ (min)*** | ***SRM 1*** | ***SRM 2*** | ***DP (V)*** | ***CE1 (eV)*** | ***CE2 (eV)*** |
| --- | --- | --- | --- | --- | --- | --- |
| *Acetamiprid* | *12.9* | *223.2 → 126.1* | *223.2 → 99.2* | *55* | *28* | *47* |
| *Ametryn* | *14.3* | *228.2 → 186.2* | *228.2 → 96.1* | *80* | *26* | *34* |
| *Amitraz* | *23.5* | *294 → 163* | *294 → 122* | *50* | *39* | *19* |
| *Atrazine* | *16.5* | *216.1 → 174* | *216.1 → 103.9* | *21* | *25* | *27* |
| *Azinphos methyl* | *18.4* | *318 → 159.9* | *318 → 132.2* | *50* | *11* | *21* |
| *Azoxystrobin* | *18.9* | *404.1 → 344* | *404.1 → 372.1* | *72* | *31* | *19* |
| *Boscalid* | *18.9* | *343.1 → 139.8* | *343.1 → 112.2* | *89* | *24* | *57* |
| *Carbaryl* | *16.3* | *202.2 → 145* | *202.2 → 127.1* | *68* | *12* | *35* |
| *Carbendazim* | *9.7* | *192.1 → 160* | *192.1 → 132.1* | *80* | *23* | *42* |
| *Carbofuran* | *15.9* | *222.1 → 123.1* | *222.1 → 165.1* | *102* | *31* | *12* |
| *Cyproconazole* | *18.0* | *292.1 → 70.2* | *292.1 → 125.1* | *16* | *35* | *35* |
| *Clomazone* | *15.9* | *240 → 125* | *240 → 99.1* | *78* | *24* | *64* |
| *Difenoconazole* | *20.8* | *406 → 251.1* | *406 → 337* | *90* | *21* | *37* |
| *Dimethoate* | *12.9* | *230.1 → 198.9* | *230.1 → 125* | *56* | *13* | *29* |
| *Epoxiconazole* | *18.6* | *330.1 → 121.3* | *330.1 → 101.2* | *36* | *27* | *63* |
| *Flutriafol* | *15.8* | *302 → 70.1* | *302 → 123.1* | *41* | *43* | *45* |
| *Flusilazole* | *19.0* | *316.1 → 247.2* | *316.1 → 163.3* | *100* | *26* | *40* |
| *Hexythiazox* | *23.3* | *353.1 → 228.1* | *353.1 → 168.1* | *70* | *23* | *34* |
| *Imazalil* | *13.8* | *297 → 255.1* | *297 → 159* | *130* | *23* | *32* |
| *Malaoxon* | *15.4* | *315.2 → 127* | *315.2 → 99* | *66* | *17* | *31* |
| *Malathion* | *19.6* | *331.2 → 284.9* | *331.2 → 99* | *56* | *11* | *35* |
| *Metalaxyl* | *16.8* | *280.2 → 220.1* | *280.2 → 192.1* | *61* | *21* | *25* |
| *Methamidophos* | *3.1* | *142 → 93.9* | *142 → 124.9* | *26* | *19* | *19* |
| *Methidathion* | *18.5* | *303 → 144.9* | *303 → 85* | *49* | *13* | *29* |
| *Methiocarb* | *18.5* | *226.2 → 169.1* | *226.2 → 121.1* | *78* | *13* | *26* |
| *Metolachlor* | *20.3* | *284.1 → 252* | *284.1 → 176.1* | *46* | *21* | *35* |
| *Metsulfuron methyl* | *11.9* | *382 → 167.1* | *382 → 141.1* | *60* | *23* | *20* |
| *Metribuzin* | *15.5* | *215.2 → 187.1* | *215.2 → 84.1* | *66* | *25* | *31* |
| *Pendimethalin* | *23.2* | *282.2 → 212* | *282.2 → 194* | *45* | *16* | *25* |
| *Penoxsulam* | *15.1* | *484 → 195.2* | *484 → 326.2* | *100* | *40* | *34* |
| *Pirimicarb* | *11.6* | *239.2 → 72* | *239 → 182.1* | *51* | *37* | *23* |
| *Pirimiphos methyl* | *21.7* | *306.1 → 108.1* | *306.1 → 164.1* | *130* | *42* | *31* |
| *Prochloraz* | *18.1* | *376 → 308* | *376 → 266* | *78* | *15* | *24* |
| *Propanil* | *17.9* | *218 → 127* | *218 → 162* | *64* | *36* | *23* |
| *Propiconazole* | *20.0* | *342.1 → 159* | *342.1 → 69.1* | *46* | *37* | *33* |
| *Pyraclostrobin* | *21.3* | *388.1 → 194.2* | *388.1 → 163.1* | *67* | *17* | *39* |
| *Pyrazosulfuron ethyl* | *18.7* | *415 → 182.2* | *415 → 139.2* | *60* | *23* | *61* |
| *Pyrimethanil* | *16.6* | *200 → 143.1* | *200 → 168.2* | *40* | *38* | *37* |
| *Tebuconazole* | *19.0* | *308.1 → 70.3* | *308.1 → 125* | *85* | *40* | *45* |
| *Thiacloprid* | *14.6* | *253.1 → 126* | *255 → 128* | *98* | *28* | *25* |
| *Thiabendazole* | *9.9; 10.2* | *202.1 → 175.1* | *202.1 → 131.1* | *100* | *34* | *43* |
| *Thiamethoxam* | *11.3* | *292 → 211.1* | *292 → 246.1* | *88* | *10* | *10* |
| *TPP (SC)* | *20.8* | *292 → 215.1* | *292 → 251.1* | *100* | *37* | *36* |
| *Tricyclazole* | *13.5* | *190 → 163.2* | *190 → 136.1* | *110* | *31* | *39* |
| *Trifloxystrobin* | *21.6* | *409.3 → 186* | *409.3 → 206.1* | *50* | *22* | *18* |

| *#* | *Pesticide* | *Mode of action* | *Molecular Formula* | *Mol. wt. (g/mol)* | *Water solubility (mg/L, 20-25 °C)* | *Vapour pressure (mPa, 20 °C)* | *log Kow* |
| --- | --- | --- | --- | --- | --- | --- | --- |
| *1* | *O-phenylphenol* | *Fungicide* | *C_12_H_10_O* | *170.2* | *1.1E6* | *9E5 (140°C)* | *3.09* |
| *2* | *Acetamiprid* | *Insecticide* | *C_10_H_11_ClN_4_* | *222.7* | *4250* | *< 1E-3* | *0.8* |
| *3* | *Ametryn* | *Herbicide* | *C_9_H_17_N_5_S* | *227.3* | *200 (pH 7.1)* | *0.365* | *2.63* |
| *4* | *Amitraz* | *Insecticide/Acaricide* | *C_19_H_23_N_3_* | *293.4* | *< 0.1* | *0.34 (25°C)* | *5.5*  *(pH 5.8)* |
| *5* | *Atrazine* | *Herbicide* | *C_8_H*14*ClN_5_* | *215.7* | *33 (pH 7)* | *3.9E-2 (25°C)* | *2.5* |
| *6* | *Azinphos methyl* | *Insecticide* | *C_10_H_12_N_3_O_3_PS_2_* | *317.3* | *28* | *5E-4* | *2.96* |
| *7* | *Azoxystrobin* | *Fungicide* | *C_22_H_17_N_3_O_5_* | *403.4* | *6.7 (pH 7)* | *1.1E-7* | *2.5* |
| *8* | *Bispyribac sodium* | *Herbicide* | *C_19_H_17_N_4_NaO_8_* | *452.4* | *6.9E4* | *5.1E-4 (25°C)* | *-1.03* |
| *9* | *Boscalid* | *Fungicide* | *C_18_H_12_Cl_2_N_2_O* | *343.2* | *4.6* | *7.2E-3* | *2.96* |
| *10* | *Bromopropylate* | *Acaricide* | *C_17_H_16_Br_2_O_3_* | *428.1* | *< 0.5* | *6.8E-3* | *5.4* |
| *111* | *Buprofezin* | *Insecticide/Acaricide* | *C_16_H_23_N_3_OS* | *305.4* | *0.387* | *4.2E-2* | *4.93*  *(pH 7)* |
| *12* | *Carbaryl* | *Insecticide/Plant growth regulator* | *C_12_H_11_NO_2_* | *201.2* | *120* | *4.1E-2* | *1.85* |
| *13* | *Carbendazim* | *Fungicide* | *C_9_H_9_N_3_O_2_* | *191.2* | *8.0 (pH 7)* | *9E-2* | *1.38*  *(pH 5)* |
| *14* | *Carbofuran* | *Insecticide/Nematicide* | *C_12_H_15_NO_3_* | *221.3* | *320* | *3.1E-2* | *1.52* |
| *15* | *Chlorfenvinphos* | *Insecticide/Acaricide* | *C_12_H_14_Cl_3_O_4_P* | *359.6* | *E-isomer 7.3*  *Z-isomer 121* | *0.53* | *E-isomer 4.22*  *Z-isomer 3.85* |
| *16* | *Chlorothalonil* | *Fungicide* | *C_8_Cl_4_N_2_* | *265.9* | *0.81* | *0.076 (25°C)* | *2.92* |
| *17* | *Chlorpyrifos* | *Insecticide* | *C_9_H_11_Cl_3_NO_3_PS* | *350.6* | *1.4* | *2.7 (25°C)* | *4.7* |
| *18* | *Chlorpyrifos methyl* | *Insecticide* | *C_7_H_7_Cl_3_NO_3_PS* | *322.5* | *2.6* | *3 (25°C)* | *4.24* |
| *19* | *Clomazone* | *Herbicide* | *C_12_H_14_ClNO_2_* | *239.7* | *1102* | *19.2 (25°C)* | *2.5* |
| *20* | *Coumaphos* | *Insecticide* | *C_14_H_16_ClO_5_PS* | *362.8* | *1.5* | *1.3E-2* | *4.13* |
| *21* | *Cyhalofop butyl* | *Herbicide* | *C_20_H_20_FNO_4_* | *357.4* | *0.44* | *5.3E-2*  *(25°C)* | *3.31* |
| *22* | *Cypermethrin* | *Insecticide* | *C_22_H_19_Cl_2_NO_3_* | *416.3* | *4E-3 pH 7* | *2E-4* | *6.6* |
| *23* | *Cyproconazole* | *Fungicide* | *C_15_H_18_ClN_3_O* | *291.8* | *93* | *2.6E-2*  *(25°C)* | *3.1* |
| *24* | *Deltamethrin* | *Insecticide* | *C_22_H_19_Br_2_NO_3_* | *505.2* | *< 2.0E4* | *1.2E-5 (25°C)* | *4.6* |
| *25* | *Diazinon* | *Insecticide/Acaricide* | *C_12_H_21_N_2_O_3_PS* | *304.3* | *60* | *12 (25°C)* | *3.3* |
| *26* | *Dicamba* | *Herbicide* | *C_8_H_6_Cl_2_O_3_* | *221.0* | *6600* | *1.67 (25°C)* | *-1.9*  *(pH 8.9)* |
| *27* | *Difenoconazole* | *Fungicide* | *C_19_H_17_Cl_2_N_3_O_3_* | *406.3* | *15* | *3.3E-5*  *(25°C)* | *4.4* |
| *28* | *Dimethoate* | *Insecticide/Acaricide* | *C_5_H_12_NO_3_PS_2_* | *229.3* | *4.0E4 (pH 7)* | *0.25 (25°C)* | *0.704* |
| *29* | *Epoxiconazole* | *Fungicide* | *C_17_H_13_ClFN_3_O* | *329.8* | *6.63* | *<0.01* | *3.33*  *(pH 7)* |
| *30* | *Ethion* | *Insecticide/Acaricide* | *C_9_H_22_O_4_P_2_S_4_* | *384.5* | *2* | *0.2 (25°C)* | *4.28* |
| *31* | *Fenhexamid* | *Fungicide* | *C_14_H_17_Cl_2_NO_2_* | *302.2* | *20 (pH 5-7)* | *4E-4* | *3.51*  *(pH 7)* |
| *32* | *Fenthion* | *Insecticide* | *C_10_H_15_O_3_PS_2_* | *278.3* | *4.2* | *0.74* | *4.84* |
| *33* | *Fenvalerate* | *Insecticide/Acaricide* | *C_25_H_22_ClNO_3_* | *419.9* | *< 0.01* | *1.9E-2* | *5.01* |
| *34* | *Fipronil* | *Insecticide* | *C_12_H_4_Cl_2_F_6_N_4_OS* | *437.2* | *1.9 (pH 5)* | *2E-3 (25°C)* | *4* |
| *35* | *Flusilazole* | *Fungicide* | *C_16_H_15_F_2_N_3_Si* | *315.4* | *45 (pH 7.8)* | *3.9E-2 (25°C)* | *3.74*  *(pH 7)* |
| *36* | *Flutriafol* | *Fungicide* | *C_16_H_13_F_2_N_3_O* | *301.3* | *130 (pH 7)* | *7.1E-6* | *2.3* |
| *37* | *Folpet* | *Fungicide* | *C_9_H_4_Cl_3_NO_2_S* | *296.6* | *0.8* | *2.1E-2 (25°C)* | *3.11* |
| *38* | *Hexythiazox* | *Acaricide* | *C_17_H_21_ClN_2_O_2_S* | *352.9* | *0.41* | *1.3E-3* | *2.75* |
| *39* | *Imazalil* | *Fungicide* | *C_14_H_14_Cl_2_N_2_O* | *297.2* | *2.6E4 (pH 4.6)* | *0.158* | *3.82*  *(pH 9.2)* |
| *40* | *Iprodione* | *Nematicide/Fungicide* | *C_13_H_13_Cl_2_N_3_O_3_* | *330.2* | *13* | *5.0E-4 (25°C)* | *3.0*  *(pH 3)* |
| *41* | *Kresoxim methyl* | *Fungicide* | *C_18_H_19_NO_4_* | *313.4* | *2* | *2.3E-3* | *3.4*  *(pH 7)* |
| *42* | *Malaoxon* | *Metabolite* | *C_10_H_19_O_7_PS* | *314.3* | *7500* | *1.3* | *0.52* |
| *43* | *Malathion* | *Insecticide/Acaricide* | *C_10_H_19_O_6_PS_2_* | *330.4* | *145* | *5.3 (30°C)* | *2.75* |
| *44* | *Metalaxyl* | *Fungicide* | *C_15_H_21_NO_4_* | *279.3* | *8400* | *0.75 (25°C)* | *1.75* |
| *45* | *Methamidophos* | *Insecticide/Acaricide* | *C_2_H_8_NO_2_PS* | *141.1* | *> 2E5* | *2.3* | *-0.8* |
| *46* | *Methidathion* | *Insecticide/Acaricide* | *C_6_H_11_N_2_O_4_PS_3_* | *302.3* | *200* | *0.25* | *2.2* |
| *47* | *Methiocarb* | *Insecticide/Acaricide* | *C_11_H_15_NO_2_S* | *225.3* | *27* | *1.5E-2* | *3.08* |
| *48* | *Metolachlor* | *Herbicide* | *C_15_H_22_ClNO_2_* | *283.8* | *488* | *4.2 (25°C)* | *2.9* |
| *49* | *Metribuzin* | *Herbicide* | *C_8_H_14_N_4_OS* | *214.3* | *1050* | *5.8E-2* | *1.6*  *(pH 5.6)* |
| *50* | *Metsulfuron methyl* | *Herbicide* | *C_14_H_15_N_5_O_6_S* | *381.4* | *548 (pH 5)* | *3.3E-7*  *(25°C)* | *-1.87*  *(pH 7)* |
| *51* | *Parathion* | *Insecticide* | *C_10_H_14_NO_5_PS* | *291.3* | *11* | *0.89* | *3.83* |
| *52* | *Parathion methyl* | *Insecticide* | *C_8_H_10_NO_5_PS* | *263.2* | *55* | *0.2* | *3* |
| *53* | *Pendimethalin* | *Herbicide* | *C_13_H_19_N_3_O_4_* | *281.3* | *0.33 (pH 7)* | *1.94 (25°C)* | *5.2* |
| *54* | *Penoxsulam* | *Herbicide* | *C_16_H_14_F_5_N_5_O_5_S* | *483.4* | *408 (pH 7)* | *9.5E-11* | *-0.354* |
| *55* | *Pirimicarb* | *Insecticide* | *C_11_H_18_N_4_O_2_* | *238.3* | *3100* | *0.43* | *1.7* |
| *56* | *Pirimiphos methyl* | *Insecticide/Acaricide* | *C_11_H_20_N_3_O_3_PS* | *305.4* | *10 (pH 7)* | *2* | *4.2* |
| *57* | *Prochloraz* | *Fungicide* | *C_15_H_16_Cl_3_N_3_O_2_* | *376.7* | *34.4* | *0.09* | *3.53*  *(pH 6.7)* |
| *58* | *Propanil* | *Herbicide* | *C_9_H_9_Cl_2_NO* | *218.1* | *130* | *0.02* | *3.3* |
| *59* | *Propiconazole* | *Fungicide* | *C_15_H_17_Cl_2_N_3_O_2_* | *342.2* | *100* | *0.56 (25°C)* | *3.72*  *(pH 6.6)* |
| *60* | *Pyraclostrobin* | *Fungicide* | *C_19_H_18_ClN_3_O_4_* | *387.8* | *1.9* | *2.6E-5* | *3.99* |
| *61* | *Pyrazosulfuron ethyl* | *Herbicide* | *C_14_H_18_N_6_O_7_S* | *414.4* | *9.76* | *4.2E-5 (25°C)* | *3.16* |
| *62* | *Pyrimethanil* | *Fungicide* | *C_12_H_13_N_3_* | *199.3* | *121 (pH 6.1)* | *2.2 (25°C)* | *2.84* |
| *63* | *Pyriproxyfen* | *Insecticide* | *C_20_H_19_NO_3_* | *321.4* | *0.68* | *<0.013 (23°C)* | *4.86*  *(pH 7)* |
| *64* | *Quinclorac* | *Herbicide* | *C_10_H_5_Cl_2_NO_2_* | *242.1* | *6.5E-2 (pH 7)* | *<0.01* | *-0.74*  *(pH 7)* |
| *65* | *Spiroxamine* | *Fungicide* | *C_18_H_35_NO_2_* | *297.5* | *Spiroxamine A (cis)- 470 (pH 7)*  *Spiroxamine B (trans)-*  *340 (pH 7)* | *Spiroxamine A (cis)- 4.0*  *Spiroxamine B (trans)-*  *5.7 (25°C)* | *A (cis)- 2.79*  *(pH 7)*  *B (trans)-*  *2.98*  *(pH 7)* |
| *66* | *τ-fluvalinate* | *Insecticide/Acaricide* | *C_26_H_22_ClF_3_N_2_O_3_* | *502.9* | *1E-3 (pH 7)* | *9E-8* | *4.26* |
| *67* | *Tebuconazole* | *Fungicide* | *C_16_H_22_ClN_3_O* | *307.8* | *36 (pH 5-9)* | *1.7E-3* | *3.7* |
| *68* | *Tetradifon* | *Acaricide* | *C_12_H_6_Cl_4_O_2_S* | *356.0* | *7.8E-2* | *9.4E-7 (25°C)* | *4.61* |
| *69* | *Thiabendazol* | *Fungicide* | *C_10_H_7_N_3_S* | *201.3* | *30 (pH 7)* | *5.3E-4 (25°C)* | *2.39*  *(pH 7)* |
| *70* | *Thiacloprid* | *Insecticide* | *C_10_H_9_ClN_4_S* | *252.7* | *185* | *3E-7* | *0.73*  *(pH 7)* |
| *71* | *Thiamethoxam* | *Insecticide* | *C_8_H_10_ClN_5_O_3_S* | *291.7* | *4100* | *6.6E-6 (25°C)* | *-0.13* |
| *72* | *Tricyclazole* | *Fungicide* | *C_9_H_7_N_3_S* | *189.2* | *596* | *5.9E-4* | *1.42* |
| *73* | *Trifloxystrobin* | *Fungicide* | *C_20_H_19_F_3_N_2_O_4_* | *408.4* | *0.61* | *3.4E-3 (25°C)* | *4.5* |
| *74* | *Trifluralin* | *Herbicide* | *C_13_H_16_F_3_N_3_O_4_* | *335.3* | *0.184 (pH 5)* | *6.1 (25°C)* | *4.83* |
| *75* | *Vinclozolin* | *Fungicide* | *C_12_H_9_Cl_2_NO_3_* | *286.1* | *2.6* | *0.13* | *3 (pH 7)* |
| *76* | *β-cyfluthrin* | *Insecticide* | *C_22_H_18_Cl_2_FNO_3_* | *434.3* | *(S)-α, (1R)-cis- + (R)-α, (1S)-cis isomers 1.9E-3*  *(S)-α, (1R)-trans- + (R)-α, (1S)-trans isomers*  *2.9E-3* | *(S)-α, (1R)-cis- + (R)-α, (1S)-cis isomers 1.4E-5*  *(S)-α, (1R)-trans- + (R)-α, (1S)-trans isomers 8.5E-5* | *5.9* |
| *77* | *λ-cyhalothrin* | *Insecticide* | *C_23_H_19_ClF_3_NO_3_* | *449.9* | *5E-3 (pH 6.5)* | *2E-4* | *7* |
| ***Internal Standard*** | | | | | | | |
| *1* | *Bromophos methyl* | *Insecticide* | *C_8_H_8_BrCl_2_O_3_PS* | *366* | *40 (25 °C)* | *17.07* | *5.21* |

*Table S3. Physicochemical properties of the selected compounds [15].*
